# Research on the construction of an urban underground parking space color system from the perspective of psychological perception

**DOI:** 10.1371/journal.pone.0313147

**Published:** 2025-01-10

**Authors:** Yuan Lv, Yanjun Shen, Boyuan Li, Jincheng Huang, Hou’an Luo

**Affiliations:** 1 College of Architecture and Civil Engineering, Xi’an University of Science and Technology, Xi’an, Shaanxi, P. R. China; 2 College of Geology and Environment, Xi’an University of Science and Technology, Xi’an, Shaanxi, P. R. China; China University of Mining and Technology, CHINA

## Abstract

Color, an intuitive element affecting human senses, can adapt to the environment of a space, evoke emotional responses, trigger and accumulate visual experiences, and enhance the effectiveness of color in shaping spatial atmosphere and reinforcing spatial divisions. In the context of rapid urban underground space development, examining the rational application of color in underground parking spaces is crucial for improving guidance, comfort, and aesthetics. This exploration is essential for achieving high-quality development in urban underground parking environments. Based on color psychology and using typical cities in China as case studies, this paper develops a color analytical framework for urban underground parking spaces through the process of “color field investigation—analysis of color influencing factors—color system construction—color application and control” and proposes a detailed chromatographic scheme. Within this framework, color field investigation serves as the foundation for determining color usage. Key influencing factors for color selection include color elements and their relationships, the type of underground parking space, and public color perception demands. The color system has been constructed including safe colors, prohibited colors, and theme colors, which has been further divided into basic, auxiliary, and accent colors. This study provides a replicable and practical theoretical and applied framework for urban underground space management and urban color planning. The framework facilitates the establishment of a standardized color system and chromatographic scheme that aligns with urban aesthetics and public psychology, thereby improving development quality and supporting the high-quality development of urban underground spaces.

## 1. Introduction

With the ongoing advancement of urbanization in China, cities increasingly encounter conflicts and pressures related to land, transportation, and population, necessitating the expansion of development space. Urban underground space has emerged as an effective solution to these issues and is increasingly favored for urban expansion [1–4]. This space significantly addresses urban land resource shortages, reduces traffic congestion, mitigates noise and air pollution, extends development areas, prevents natural disasters, and supports sustainable urban development [5–10]. Underground space can be viewed as the ultimate boundary of a city [11], with its uncontrolled development and design affecting or limiting its utilization. Loretta von der Tann introduced the concept of “sustainable underground urbanism” (SUU), aimed at understanding the contributions of various disciplines to sustainable urban development and enhancing the urban living experience [12, 13]. Thus, the design of urban underground space should be considered alongside surface space, particularly in shallow underground areas [14], with interventions made judiciously to ensure livability and human development [9].

Compared to urban outdoor spaces, urban underground spaces exhibit notable differences in both objective physical conditions and subjective psychological feelings [15]. These spaces often lack natural elements such as daylight, fresh air, and greenery, and are typically characterized by high humidity and noise, which complicates evacuation. Consequently, urban underground spaces are often dark, enclosed, and visually uninviting. Such physical conditions can lead to subjective psychological discomfort, impairing orientation recognition and reducing environmental awareness, which can result in negative perceptions and resistance to underground spaces [16]. Therefore, it is essential to investigate methods to mitigate these negative psychological effects.

As a crucial medium for interaction between individuals and their environment, color can influence emotional and psychological states and is a key factor affecting human senses. The scientific and rational application of color in underground spaces can enhance visual appeal and ameliorate dark and claustrophobic conditions, leading to positive emotional and psychological effects [17–19]. Effective use of color can improve spatial ambiance, strengthen spatial delineation, optimize underground space quality, and enhance public comfort, thereby integrating color psychology with behavioral principles and color application. When the color of underground spaces is regarded as part of the urban landscape, its true value becomes evident [20].

Among various types of underground spaces, underground parking lots are relatively large in scale. The “Nanjing Underground Space Development Report” indicates that by the end of 2020, the total underground space in Nanjing had reached 71.9 million square meters, with 45.9 million square meters allocated to underground parking facilities, representing 63.87% of the total. Nanjing reflects a broader trend in the development and utilization of urban underground spaces across China. According to the 2023 Blue Book of Urban Underground Space Development in China, the scale of urban underground parking space is substantial, with the underground parking rate—the proportion of underground parking spaces relative to the total number of urban parking spaces—exceeding 30% in cities such as Hangzhou, Beijing, Shanghai, Wuhan, Shenzhen, Tianjin, and Guangzhou [21]. Additionally, policies, regulations, and normative documents related to underground parking constitute 24% of all underground space documentation [22]. The extensive construction of underground parking facilities also highlights the significant public demand for such spaces for work and daily activities.

Therefore, it is essential to investigate the application of color in underground parking spaces through the lens of color psychology. This includes developing an analytical framework for color application and offering a reproducible and scalable chromatographic scheme. Such exploration is necessary to address the dual effects of color in enhancing the aesthetic appeal of underground spaces and mitigating negative psychological responses.

## 2. Literature review and analytical framework

### 2.1 Literature review

The application of color in urban settings encompasses both aboveground and underground spaces. Due to the earlier development and larger scale of aboveground spaces, there has been substantial research on color application in these areas. Currently, urban color research primarily addresses color theory in aboveground spaces [23, 24] and the use of color in cities, buildings, signage, and other areas [25–30]. This includes overall urban color planning [4, 31], building and street facades [32–34], and urban landscape color [35]. Color has become a crucial factor in contemporary research and practice related to the planning of urban aboveground spaces.

In contrast, research on the application of color in urban underground spaces is relatively limited, with existing studies primarily focusing on logos, guide systems, station spaces, subway lines, and interior colors of subway environments. Scholars have addressed issues such as visual confusion, color pollution, inconsistency in sign colors, and ineffective guide colors in subway transfer areas in various cities (e.g., Paris, Tokyo, New York, Shanghai, Beijing, Guangzhou) by proposing relevant logos and guide color planning schemes and countermeasures [17, 36–41]. Additionally, the color design of traffic systems, considering the selection of colors for carriages and lines, has been explored from the perspective of overall coherence with urban characteristics [42, 43].

Urban underground spaces include not only subway systems but also underground parking lots, pipeline corridors, commercial facilities, public service facilities, and more. Research on underground parking planning has received significant attention, focusing on aspects such as graphic design, engineering design, layout, and management [44–46]. However, studies on the application of color in urban underground parking lots remain scarce. Scholars have explored the application of color in these spaces from various angles, such as considering the characteristics of components like parking spaces, guide spaces, and ground pavements, and proposing color use principles for each component at a macroscopic level [47]. Additionally, research has summarized the roles that color can play in underground parking lots [47, 48] and discussed color function and application from the perspective of visual sensory design [49–52], including recommendations for color selection.

The aforementioned research highlights that, in the quest for high-quality urban spaces, underground parking lots should meet basic standards for creating an inviting public environment. While the importance of color application in underground parking lots is widely acknowledged, existing studies have not fully demonstrated the specific impact of color application on these spaces from the perspective of color psychology.

In conclusion, the existing research provides significant reference value for comprehending the practical application of color in underground spaces. However, several notable limitations require additional refinement:

1. Research has primarily concentrated on color application within subway station environments, with relatively limited in-depth analysis of the substantial area occupied by underground parking facilities.
2. From a research perspective, the analysis has predominantly concentrated on composition, color function, and visual sensory requirements within underground parking lots. However, it is essential to acknowledge that colors impact users through visual sensory perception within various spatial compositions and can induce psychological changes. This shift in psychological response to colors is central to human-centered design and thus merits investigation from a perspective grounded in color psychology.
3. In terms of content, the focus has been on discussing color functions and principles, while specific design effects have received less attention. Additionally, a comprehensive system for color and chromatographic scheme libraries is absent.

In light of this, from the standpoint of color psychology, urban underground parking lots are the focus of this research. A comprehensive analysis of users’ essential needs regarding color use in underground environments is carried out, and the current state and challenges related to color application in these spaces are outlined. Additionally, an analytical framework and chromatographic scheme for the color system of urban underground parking facilities are proposed. The objective is to present a theoretical and practical framework that establishes a standardized method for color application in urban underground parking areas.

### 2.2 Theoretical basis

#### 2.2.1 Color theory

**Fig 1:**
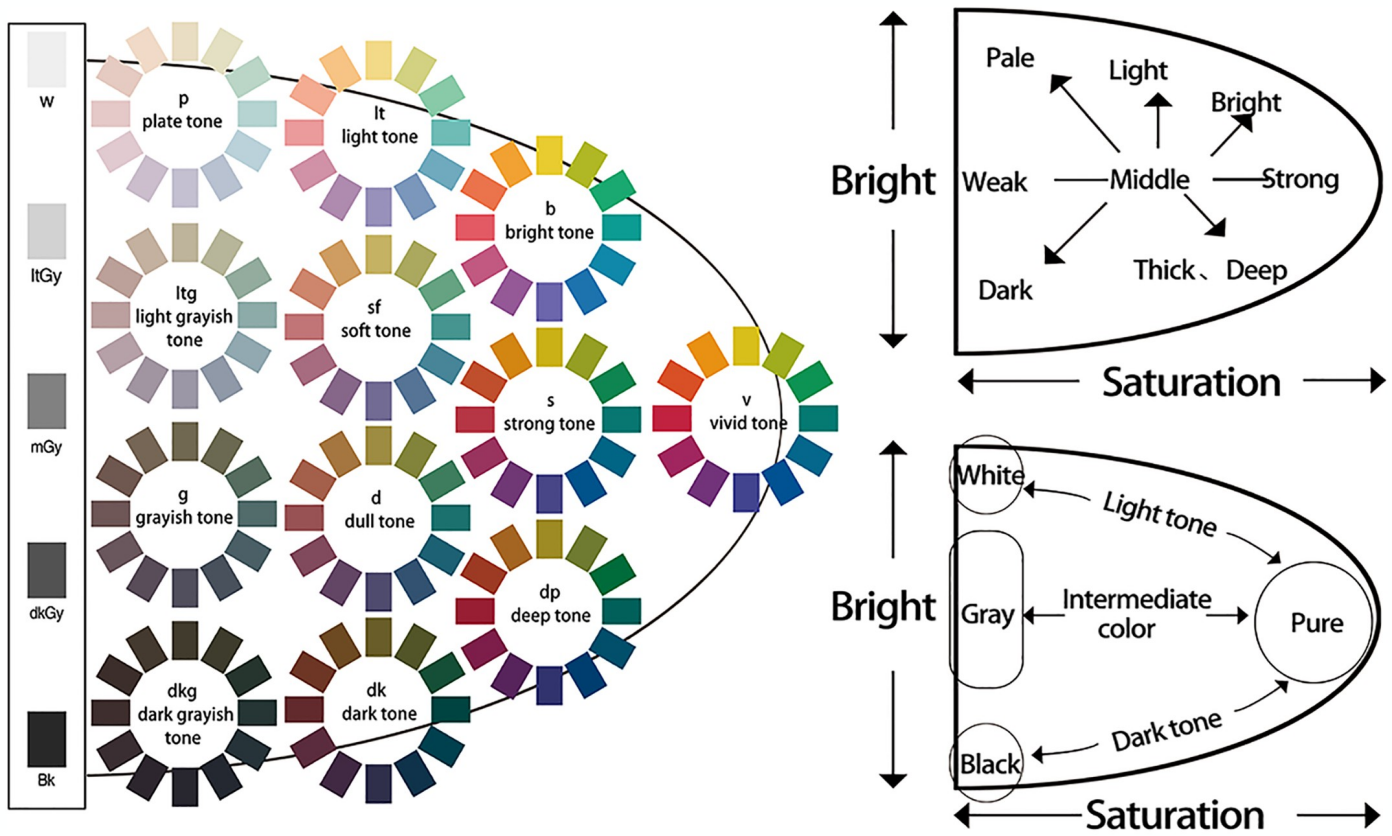
Color system of urban underground space—PCCS. Source of the image: https://www.photophoto.cn/sucai/15945039.html.

The color scheme for urban underground parking spaces is based on the Japanese Practical Color Coordinate System (PCCS). Created by the Color Research Institute of Japan, this practical color matching system incorporates elements from both the Munsell color system and the Ostwald color system [53]. Depicted by a hue ring, it includes a wide range of color types with significant contrast, establishing it as a well-recognized theoretical framework. The PCCS is mainly employed in urban color planning and environmental color research (Fig 1).

#### 2.2.2 Color psychology

Color is not only an essential method for conveying information but also has the capacity to affect psychological processes. Color psychology is a field that investigates the general principles of how colors are perceived and interpreted by individuals and society. It suggests that colors allow for the identification of objects in the physical world and can subsequently influence perception, memory, associations, and emotions. The meaning and use of various colors within a particular geographic area are determined by collective agreement and established regulations.

From the perspective of color psychology, the concepts of “tonality” and “mode” from music can be applied to understand the psychological impacts of color expression. “Tonality” pertains to various colors, reflecting an overall color style, while “mode” refers to color composition, indicating the harmony achieved through color combinations. The interaction of these concepts illustrates individuals’ psychological preferences for color perception. In this context, it is crucial to consider the selection angle of colors on the color wheel when achieving color harmony. This can involve using monochromatic schemes with varying lightness and chroma within the same hue spectrum or contrasting schemes with significant hue differences. Furthermore, the area size where colors are applied influences their psychological effects.

Therefore, according to the theory of color psychology, color depends on visual perception, which subsequently stimulates the cognitive understanding of space for the observer. Color significantly influences spatial recognition and has the ability to convey emotions beyond what physical or morphological elements alone can express [54]. The strategic application of color can help modify public perceptions of underground spaces, thereby addressing psychological barriers and doubts, and ultimately improving their use and appreciation of urban underground areas.

#### 2.2.3 Golden section principle

In the use of color in urban underground parking spaces, relying on a single color greatly reduces the visual appeal. Hence, it is important to incorporate multiple colors. The difficulty lies in coordinating their proportions effectively. The golden section is a valuable tool for achieving balanced proportions. This mathematical ratio, about 0.618, has been supported by extensive research and psychological studies [55–59] as providing an aesthetically pleasing and harmonious proportional relationship.

The precise proportions, artistic quality, and harmony inherent in the Golden Section possess significant aesthetic value. This principle is evident in numerous masterpieces, including the Pyramids of Egypt, the Parthenon in Athens, Greece, and the Taj Mahal in India, all of which are renowned for incorporating the Golden Section in their design.

Therefore, this paper incorporates the concept of the Golden Section ratio into the study of theme color to improve the visual appeal of color application in urban underground spaces.

### 2.3 Construction of a color system analytical framework for underground parking spaces on the basis of color psychological perception

**Fig 2:**
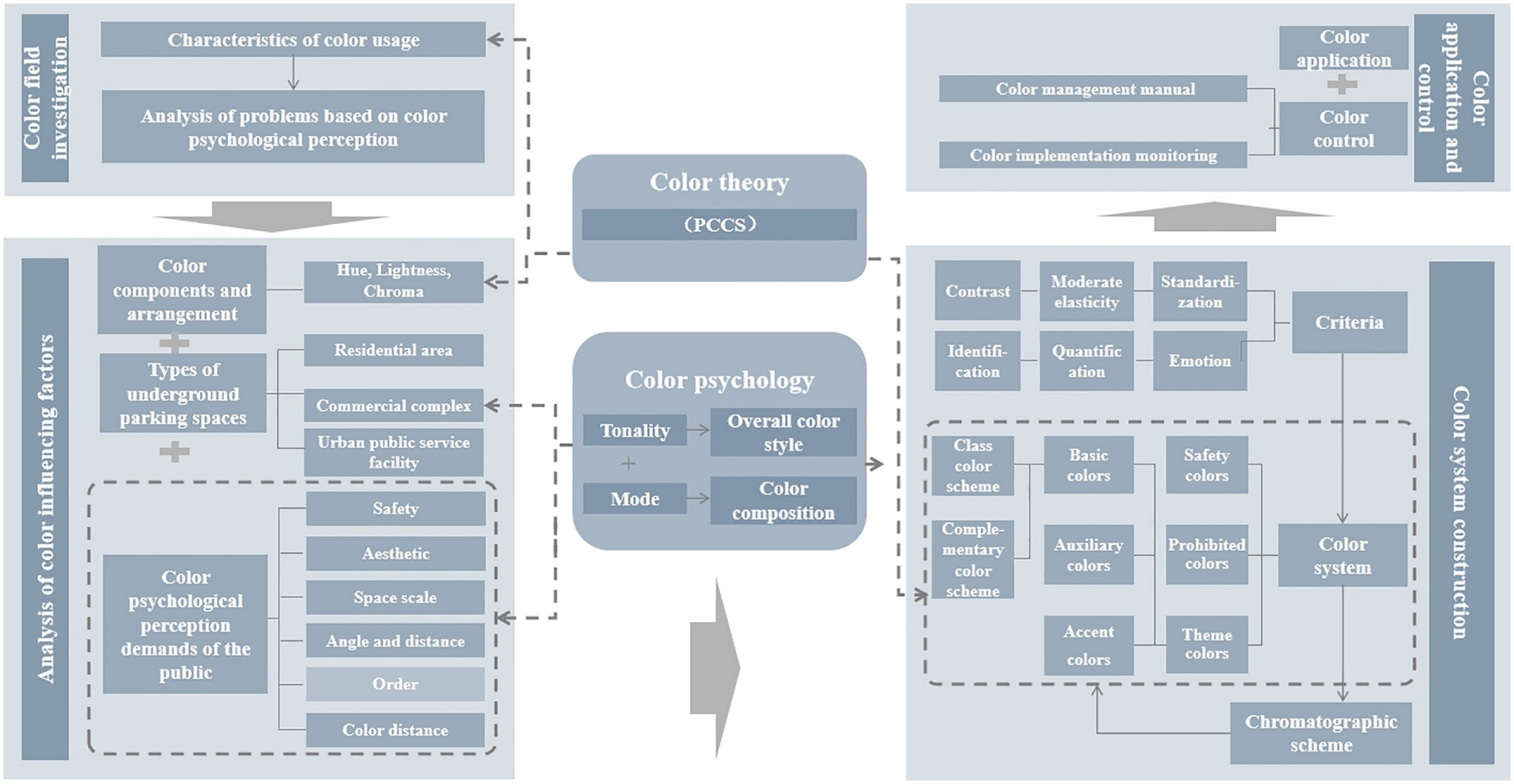
Analytical framework for color systems of underground parking spaces based on color psychological perception.

Based on a thorough review of relevant literature, theoretical background, and field investigation, the research emphasizes the critical need to develop a color system for urban underground parking spaces grounded in color psychology perceptions. The analytical framework of this system is depicted in Fig 2 and includes four essential components: “color field investigation—analysis of color influencing factors—color system construction—color application and control.”

First, color samples are gathered from urban underground parking spaces through field investigations, summarizing the characteristics of color usage and identifying existing issues in color application. Second, it involves examining the factors that influence the development of a color system to establish a basis for its design. Third, a color system is constructed based on the identified issues and influencing factors. Finally, the goal is to apply the results and implement effective color control measures.

#### 2.3.1 Color field investigation

Understanding the color of urban underground spaces requires acquiring color perception through public participation in color research. By involving the public in a two-way interaction with the colors of underground spaces, a perceptual image of urban underground parking colors is formed, which subsequently helps to refine the characteristics of color usage.

The analysis of problems based on color psychological perception involves summarizing the current situation and identifying issues in color application within urban underground parking spaces. The goal is to offer guidance for developing an effective color system, which is essential for researching color system application in underground parking spaces from a color psychology perspective.

#### 2.3.2 Analysis of color influencing factors

*(1) Color elements and their relationships*. The clarity of psychological color perception is influenced by the prominence of color features. The color characteristics of urban underground spaces are conveyed through the attributes, tonal relationships, and areas of different color carriers.

In evaluating color attributes and tone relationships, attention must be given to the multidimensional aspects of hue, lightness, chroma, and the contrast between varying levels of lightness and chroma. Bright colors can enhance clarity and evoke a sense of brightness, while dark colors might impart a heavy or somber impression. High-intensity colors are capable of creating a vibrant and intense effect, whereas low-intensity colors may suggest a more subdued and tranquil perception.

To assess the use area of color carriers, the primary-secondary relationships between different colors can be determined by comparing their proportional area relationships. Furthermore, addressing any conflicting interactions arising from the balanced application of colors aids in achieving a more harmonious overall appearance of the space.

*(2) Types of underground parking spaces*. Color selection should take into account the type of urban underground parking space. Underground parking can be divided into three types: residential underground parking, commercial complex underground parking, and urban public service facility underground parking. Given that underground parking accommodates both vehicles and pedestrians, safety is the primary consideration for color choices. Moreover, the color scheme should assist in quick vehicle positioning and provide clear indications of entrances within these facilities.

Based on these considerations, the underground parking facility in a residential area should focus on enhancing the visibility and convenience of the building’s entrances and exits. The color scheme should create an atmosphere of warmth and comfort for users. Conversely, underground parking areas in commercial complexes frequently use bold advertising designs to draw attention to their products and services. While these colors are intended to attract attention, they should not interfere with safety and guidance within the facility; thus, softer and more refined colors might be more appropriate. Public service facilities, including hospitals, libraries, museums, and gymnasiums, should select colors that convey stability and calm, reflecting the character of their institutions.

*(3) Color psychological perception demands of the public*. The psychological perception of color is influenced by the visual experiences of individuals using urban underground spaces. The significance of addressing public preferences for color in these areas is crucial. This study explores public demands related to color perception from various perspectives:

1. Safety perception indicates that claustrophobia, darkness, poor visibility, and inadequate guidance in underground spaces can greatly reduce users’ sense of safety and increase feelings of depression, fear, and anxiety. Thus, it is essential to improve color guidance through thoughtful selection, signage system implementation, and emergency facility arrangements, emphasizing color schemes that can help alleviate users’ discomfort [60].
2. Aesthetic perception requires that the primary use of color in underground spaces meets users’ aesthetic preferences. Given the broad range of individual color preferences, it is important to choose colors that are visually appealing while being rational and consistent with public aesthetic expectations.
3. Space scale perception demands: Users entering underground spaces need to form an impression of its scale. Smaller underground spaces typically create a uniform feeling and image through color, while larger spaces benefit from distinct color partitions that help in shaping perceptions of the space’s scale.
4. Angle and distance perception demands: Different angles and distances can affect color perception, leading to variations in unity, balance, appropriateness, proportionality, and security among users. Additionally, the interactions between cold and warm colors [61], light and dark contrasts, color contrasts, and area contrasts can influence aspects such as depth perception, distance perception, and size perception.
5. Order perception demands: The natural world exhibits a degree of order in the variation and connection of brightness, intensity, temperature, grayscale, light and shadow, and color [62]. In underground spaces, users rely on easily interpretable color sequences to establish a sense of spatial order. By employing varied and layered color combinations, underground spaces can create a cohesive and overall sense of orderly perception [63], reflecting the rhythm and appeal of organized color arrangement.
6. Color distance perception demands: The differences in function, direction, and volume of underground space are perceived by users through color, which is expressed in terms of color distance contrast. A larger color distance results in a more pronounced contrast between colors [64].

#### 2.3.3 Color system construction

**Fig 3:**
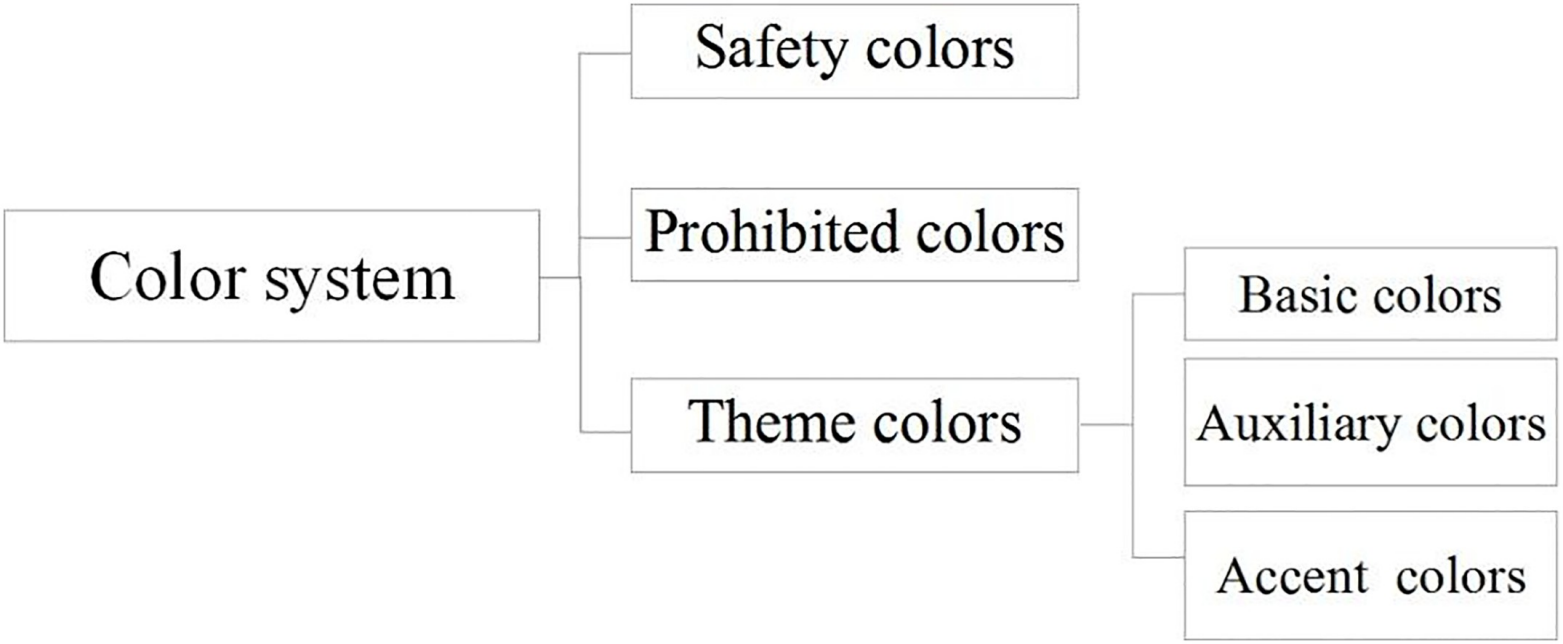
Color system.

The application of color in urban underground parking spaces should encompass ground surfaces, top surfaces, column surfaces, signage, pipelines, and other various elements. The choice of color is influenced by the functions of different elements. A systematic framework is required to accommodate multiple spaces and diverse colors. Accordingly, this study proposes a color system for urban underground parking spaces (Fig 3), which includes safety colors, prohibited colors, and theme colors. Safety colors are those that convey safety-related information; prohibited colors are those deemed unsuitable for use in urban underground parking spaces based on safety, identification, aesthetics, and other functional requirements; and theme colors are color combinations developed to meet public color psychological demands, based on established safety and prohibited colors. Theme colors are categorized into basic colors, auxiliary colors, and accent colors. The color scheme for urban underground parking areas should be established within this color system framework.

The development of the chromatographic scheme for urban underground parking spaces should meet the following criteria: 1) Contrast: Color contrast should be employed to highlight the color carriers and their functions, aiding in the differentiation of information conveyed by different colors. 2) Moderate Elasticity: The chromatographic design should be adapted to the scale, lighting, and type of business of the underground space, providing a selection of options with moderate elasticity. 3) Standardization: Adherence to national standards for color indications of prohibition and warnings is essential. This ensures the consistent and standardized use of colors for specific areas and symbols, addressing safety, guidance, and comfort needs. 4) Identification: Color choices should have clear identification properties. Chaotic color combinations should be avoided to improve user orientation and reduce disorientation or confusion. 5) Quantification: Utilizing quantified color schemes should replace subjective designer decisions. This approach ensures that design intentions are accurately conveyed and user expectations are met, with RGBs possibly used to represent specific colors. 6) Emotion: To alleviate negative emotions linked with dark underground environments, color choices should produce positive emotional and physiological effects, reducing visual misjudgments and accident risks.

#### 2.3.4 Color application and control

The creation of a color system and the formulation of a color scheme are designed to offer a foundation and guidelines for color selection in underground parking spaces, aiming for practical application. Consequently, parking management personnel can utilize the color scheme to focus on zoning and fully utilizing the multiple functions of colors. Additionally, developing a color management manual with various schemes can assist management personnel in daily operations and facilitate communication and coordination with the construction entity, thereby effectively organizing the color environment. This approach enhances user satisfaction in both visual and psychological aspects, contributing significantly to the overall quality of urban underground spaces.

## 3. Research object and data

### 3.1 Research object

As stated in the introduction, urban underground parking spaces occupy a substantial portion of underground spaces and play a crucial role in supporting aboveground urban activities, including residential areas, commercial complexes, and public service facilities. These parking spaces are closely linked to the daily lives and activities of the public. Consequently, this study focuses on urban underground parking spaces to investigate spatial color application issues and offer recommendations for improving the quality of the underground space environment.

### 3.2 Data sources

**Fig 4:**
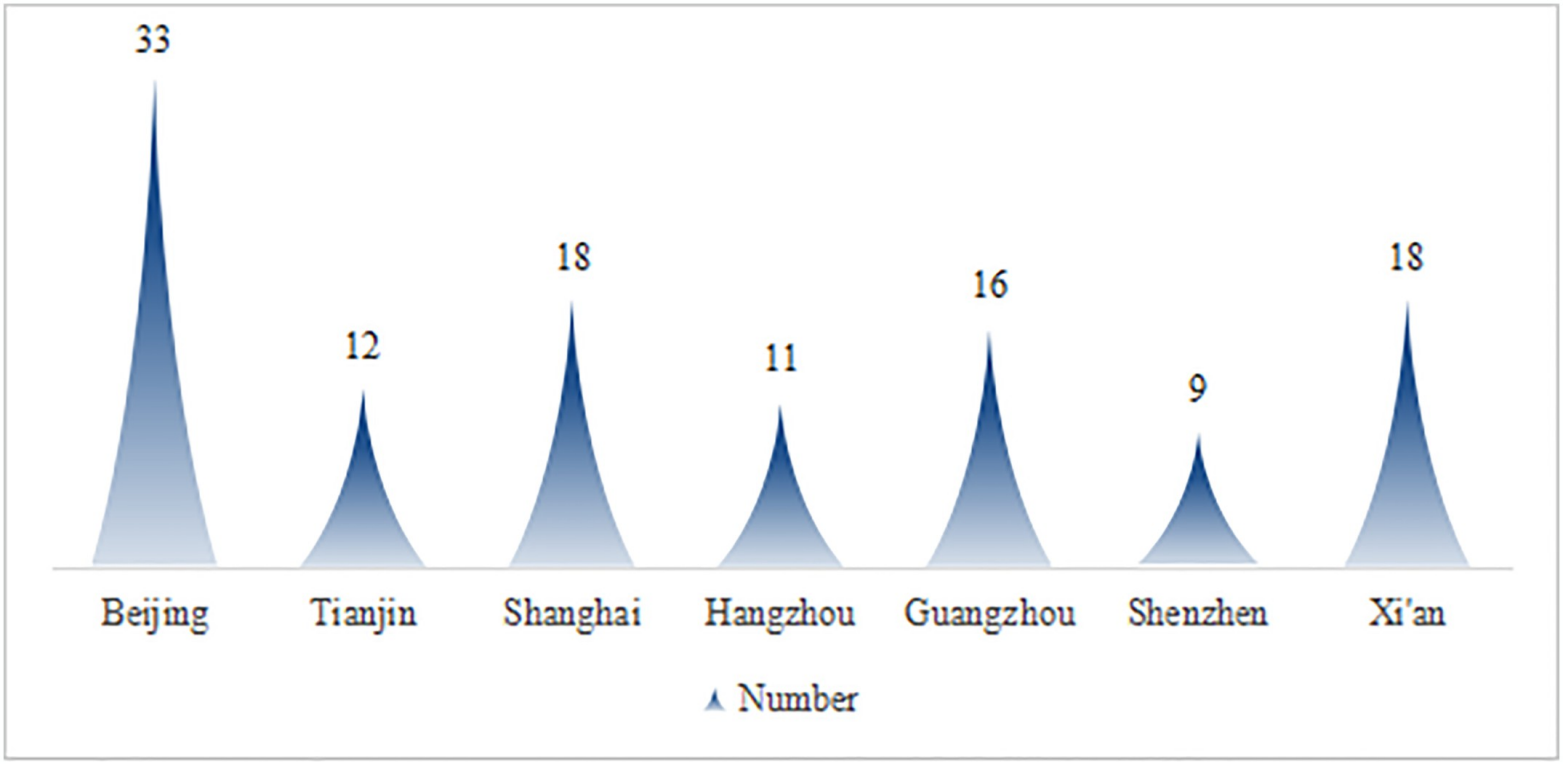
Number of survey points for underground parking spaces in each city.

To achieve a thorough understanding of the current use of color in urban underground environments, field investigations were conducted on underground parking facilities across several major domestic cities (including Beijing, Tianjin, Shanghai, Hangzhou, Guangzhou, Shenzhen, and Xi’an). The collected sample data encompassed various types of urban underground parking spaces, including commercial complexes, public service facilities, and residential areas. The quantities surveyed in each city are illustrated in Fig 4. Furthermore, both structured and unstructured interview techniques were extensively utilized to gain insights into users’ perceptions, needs, and recommendations concerning the application of color in these underground spaces.

## 4. Research results

### 4.1 Color demand level: Research on public color perception

**Fig 5:**
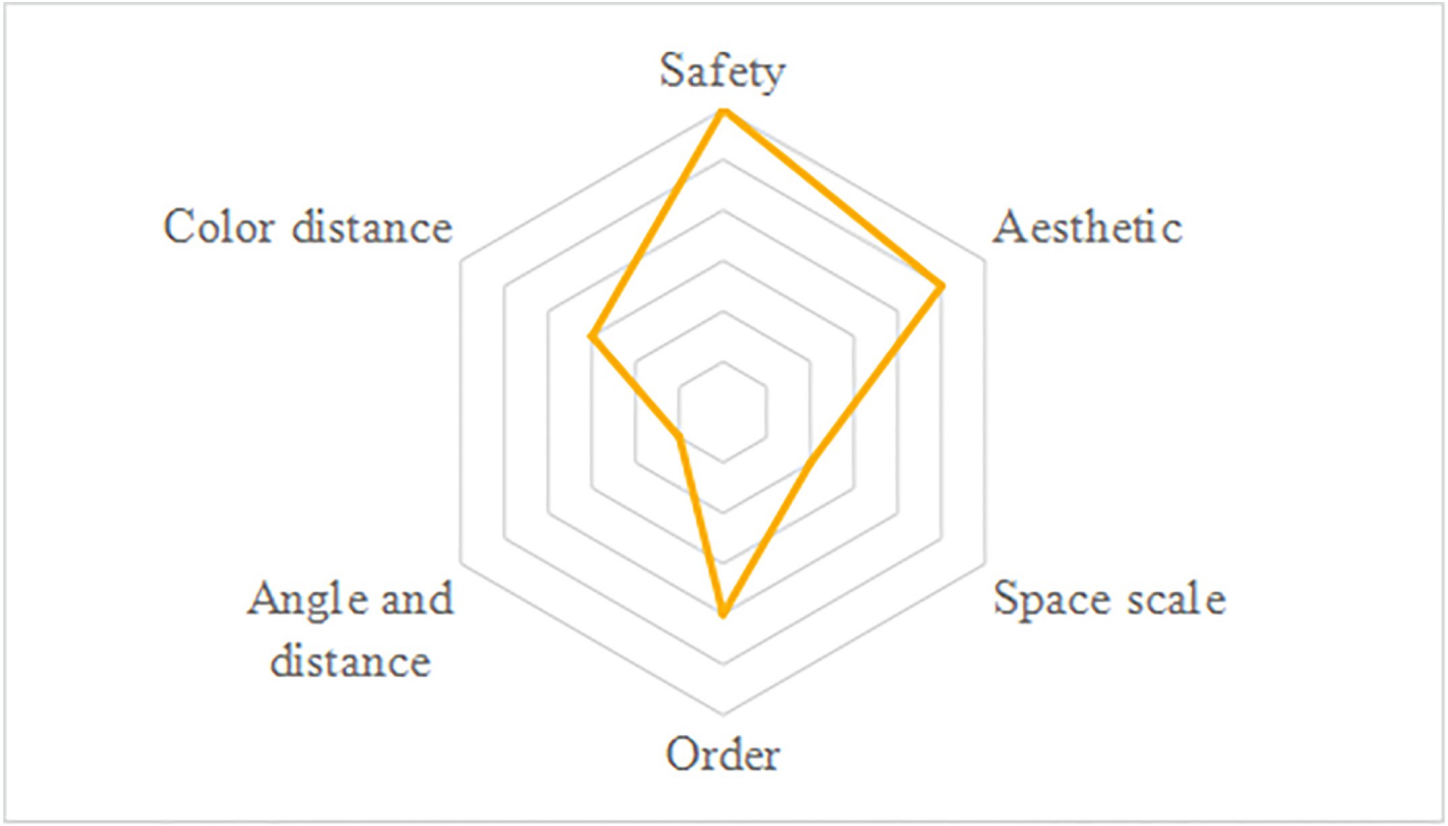
Order of public perception demands.

As outlined in the previous analytical framework, public psychological perception of color requirements is a significant factor influencing color selection. To assess this in major cities, an extensive public participation survey was conducted, addressing six primary demands: safety perception, aesthetic perception, spatial scale perception, angle and distance perception, order perception, and color distance perception. The emphasis was placed on identifying the key demands and their hierarchical order concerning color application in urban underground parking spaces (Fig 5).

Public surveys indicate that safety is the primary concern for the public. It is believed that conveying safety through color design involves creating safety-related signage in accordance with relevant regulations. Aesthetic demands are ranked second in importance. This suggests that the public not only seeks quality in private environments but also has higher expectations for public spaces, such as underground parking facilities.

The overall color perception of underground parking spaces is influenced by the sequence of colors based on public opinion. A more defined color order enhances the sense of cohesion. Coordinated and transitional color schemes can offer dual visual and psychological satisfaction while contributing to a perceived sense of spatial dimension.

Color distance is represented as a measure of the degree of uniformity in space color differences. From the perspective of color distance, whether the distances between similar or contrasting colors are smaller or larger, they must still fulfill identification objectives.

The least attention from the public is given to angle and distance perceptions, indicating a belief that variations in angle and distance do not impact their recognition of colors within underground spaces.

### 4.2 Color supply level: Sorting out the status quo of color application

The appropriate use of color is essential for ensuring a comfortable, visually appealing, and functional experience in underground parking areas. A review and summary of investigating findings from standard urban underground parking facilities in China reveal the following issues concerning the application of colors in these spaces from a psychological perspective:

#### 4.2.1 Confusion in color applications

**Fig 6:**
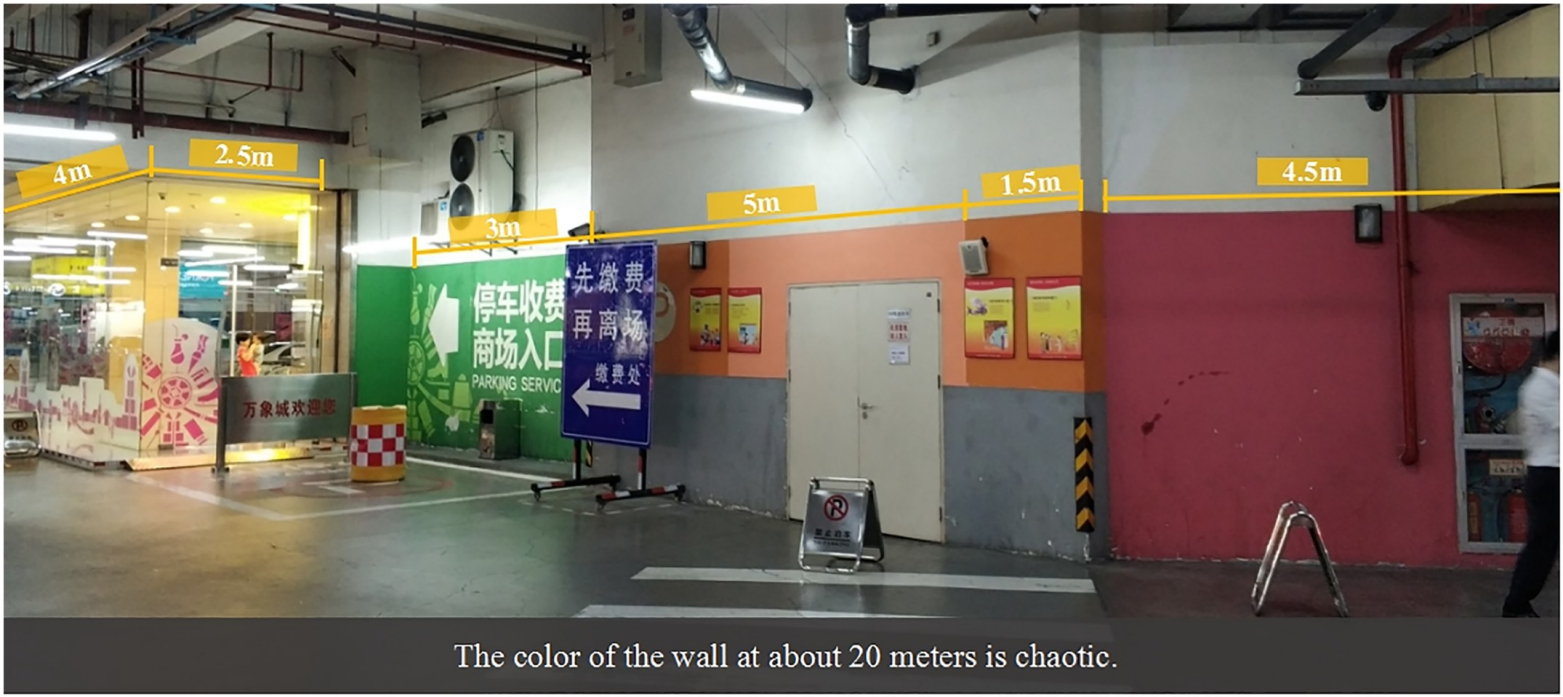
Confusion of color application (Hangzhou MIXC underground parking lot). (Photo credit: Photo by the author).

The public perceives the application of colors in some urban underground parking spaces as disordered, with a lack of overall coordination and uneven color implementation. This situation complicates positioning and identification, particularly due to the indiscriminate use of colors in facilities and commercial advertisements, which significantly disrupts the sense of order and visual appeal, resulting in systematic color deficiencies. For instance, the Hangzhou MIXC underground parking lot displays a variety of colors on the same wall, creating a chaotic visual effect (Fig 6). The survey indicated that the public prefers a systematic color scheme to enhance their psychological experience, such as aesthetics and comfort.

#### 4.2.2 Insufficient color guidance functionality

**Fig 7:**
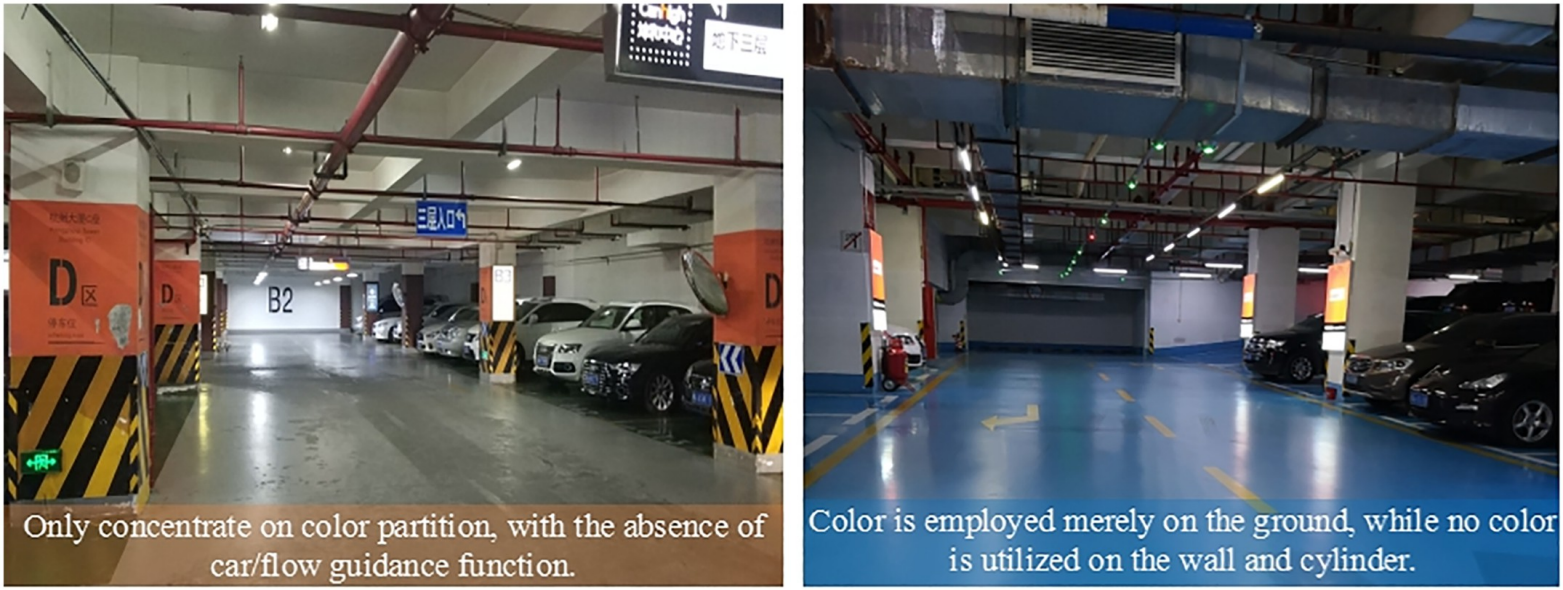
Lack of color guidance function (Photo credit: Photo by the author). (a) Guangzhou Zhengjia Square underground parking lot, (b) Hangzhou Kunhe Center underground parking lot.

Certain underground spaces prioritize color zoning by employing various colors to segment the area but fail to consider color’s role in directing pedestrian and vehicular movement. There is an absence of standardized color guidance systems, and color usage is often inadequate. For example, our investigation showed that some underground spaces apply color only to the ground or cylindrical surfaces, which does not effectively guide or position people and vehicles (Fig 7). Feedback from the public indicated a preference for familiar color sequences, such as “red-orange-yellow-green-blue-violet” and “A-B-C-D-E…” for zoning layouts. This order not only aids in positioning and guidance within zones but also helps individuals perceive the space’s scale relative to their position. Implementing these suggestions can remedy deficiencies in color zoning and enhance guidance in underground spaces.

#### 4.2.3 Unclear color labeling in specific areas

**Fig 8:**
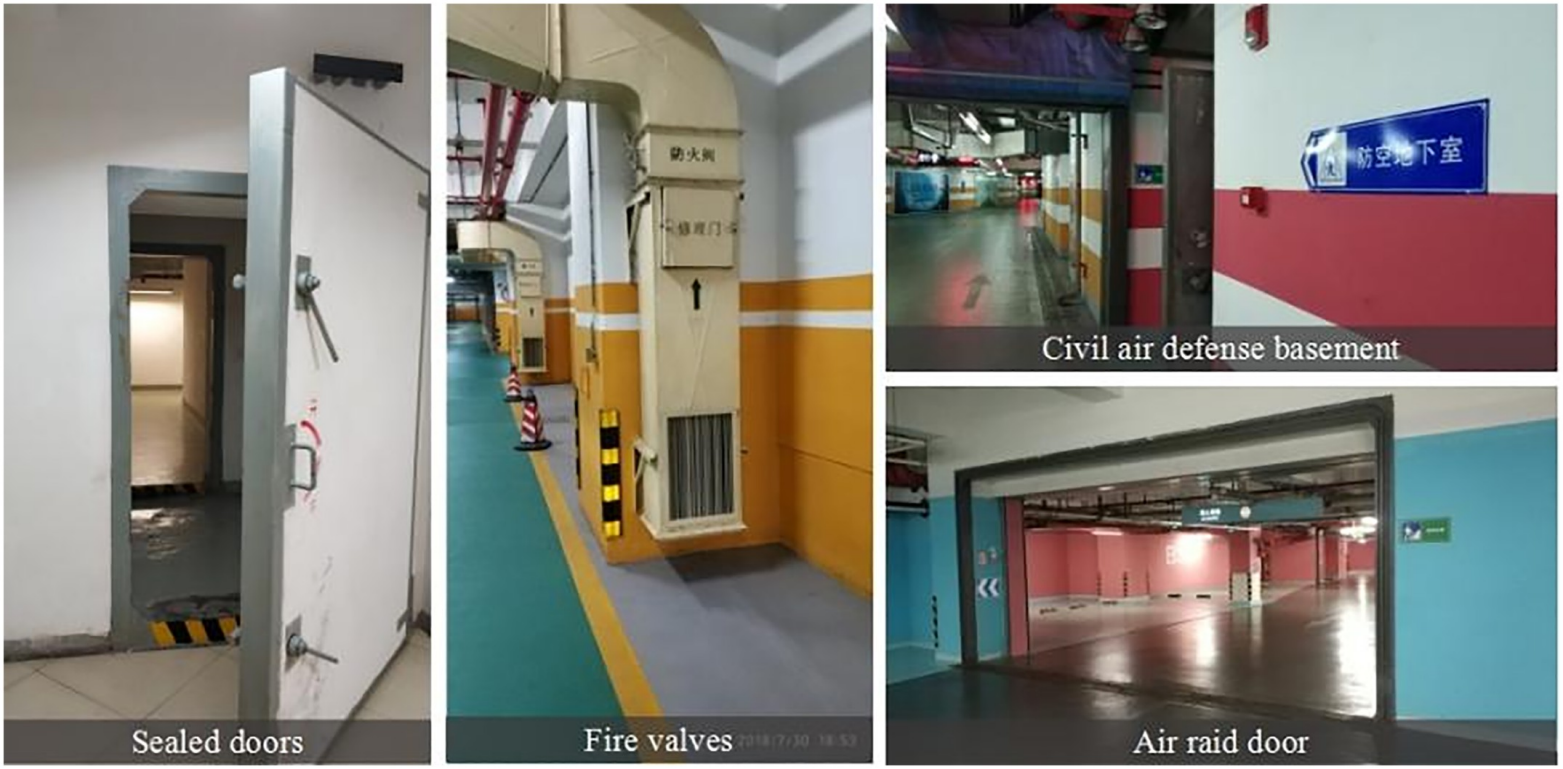
Unclear color marks in special locations (Photo credit: Photo by the author).

**Fig 9:**
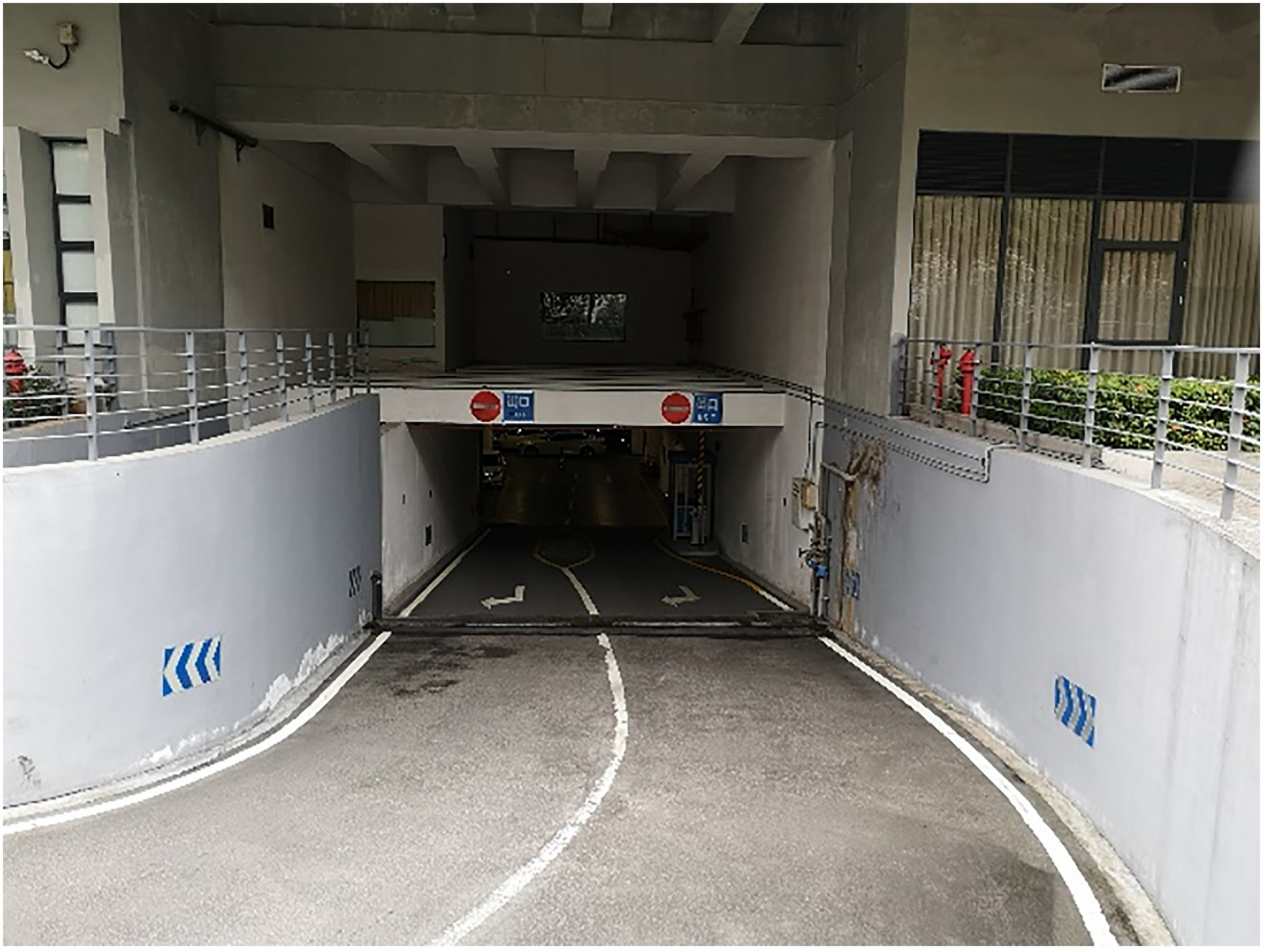
Lack of color connections (Photo credit: Photo by the author).

Signage in some underground spaces faces issues such as insufficient use of color, confusion due to cluttered commercial advertisements, and inadequate emphasis on warnings and key information. These problems decrease the effectiveness of signs in spatial orientation and guidance. For instance, signage at critical locations, such as sealed doors, fire valves, and civil air defense facilities, often lacks visibility, creating significant security risks (Fig 8). Additionally, color usage for guidance in aboveground connection areas is inadequate. Some of these areas either do not use color or lack cohesive color schemes, as seen in the connection between the underground garage and the aboveground at the Guangdong Provincial Museum (Fig 9). Public interviews revealed a strong belief in the importance of clear signage for guidance and warnings in underground spaces. A standardized color scheme is necessary to improve the effectiveness of these signs.

## 5. Discussion

### 5.1 Development of a chromatographic scheme for urban underground parking spaces based on color systems

The research results suggest that the application of color in underground parking spaces suffers from an inadequate supply of colors, which fails to address the diverse needs of public usage. Additionally, it does not accommodate the varying requirements of individuals using these spaces. Unlike earlier studies that concentrated mainly on the macroscopic functionality [48] of underground parking areas, the influence of color application appears to be relatively minimal. The absence of a comprehensive chromatographic scheme [47] limits both the visual impact and theoretical discussion. Hence, it is recommended that, based on the color system analytical framework outlined in section 2.3 of this study, the development of a set of chromatographic schemes aligned with the established color system is crucial. This approach will lay the groundwork for effective implementation in the management of underground parking spaces.

#### 5.1.1 Proportion of color area

**Fig 10:**
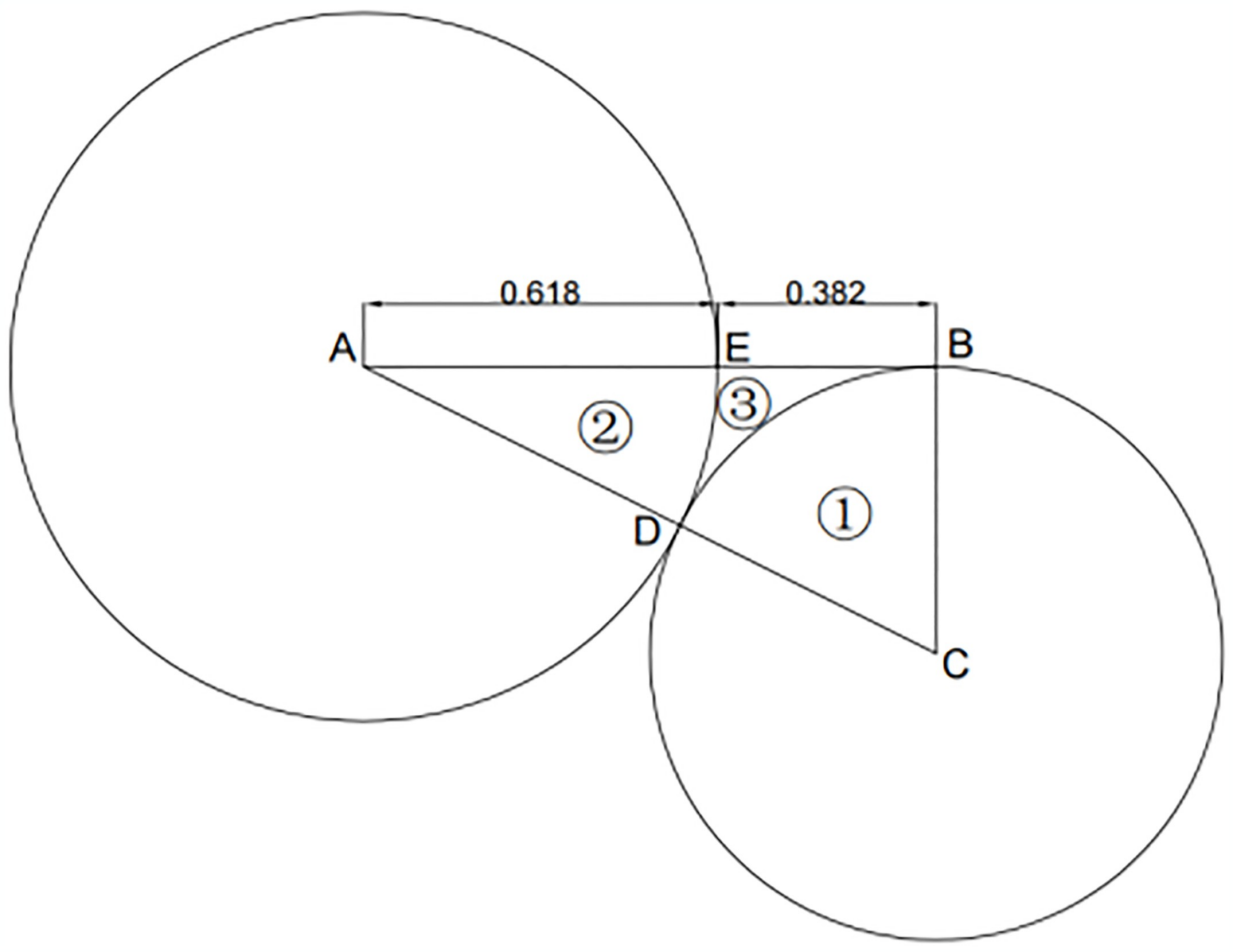
Color area ratio based on the golden section ratio.

In the earlier section of this study, it was consistently highlighted that the composition of color, especially the proportional distribution of color areas, warrants attention. By applying the golden section proportion and the theme color composition from the color system, the proportional relationships of color areas were derived. The derivation process is as follows: initially, draw line segment AB and locate the golden section point E on this segment; then, from point B, draw a perpendicular line segment BC to AB, with BC equal to 1/2AB, and connect AC. Subsequently, with A and C as the centers, draw two circles with radii AE and BC, respectively, ensuring they are tangent at point D. This process divides ΔABC into three regions: sector BCD (denoted by symbol ①), sector ADE (denoted by symbol ②), and the irregular shape BDE (denoted by symbol ③) (refer to Fig 10). Calculations show that the area proportions of these regions are 55.3%, 35.4%, and 9.3%, respectively. This proportional relationship is then applied to the area distribution of the three colors in the theme: basic colors, auxiliary colors, and accent colors.

#### 5.1.2 Chromatographic determination

Based on the color system established in section 2.3.3, the detailed chromatographic scheme for safe colors, prohibited colors, and theme colors in this study is outlined as follows:

*(1) Safety colors*. According to the national standard “Safety Color” (GB2893-2008), safety colors include red, yellow, blue, and green (Table 1). These colors are used for safety warning signs and are governed by strict regulations concerning both their shape and color. The application of these standardized safety colors is intended to regulate and guide public safety behavior in various environments. When utilizing safety colors, it is crucial to select a contrasting color to effectively emphasize the safety color.

**Table 1:** Overview of safety color information in urban underground space.

Color	Color Blocks	RGB	Meaning
Red		R:255 G:0 B:0	No, Stop, danger, fire facilities
Yellow		R:255 G:255 B:0	Warnings, attention
Blue		R:0 G:0 B:255	Instruction, compliance
Green		R:0 G:255 B:0	Safe, pass, tips

*(2) Prohibited colors*. Prohibited colors are primarily achromatic. Achromatic colors are those that lack hue, including black, white, and various shades of gray. These gray shades are defined by equal values for the R, G, and B color channels within the range of 0–255. When all three values are set at 128, the color produced is neutral gray, which can be further classified as light or dark (Table 2). The use of dark gray and black with low brightness can elicit feelings of depression, coldness, and confinement in individuals. Additionally, these colors may impair visibility in underground spaces. Therefore, black and dark gray color schemes should be avoided in underground environments.

**Table 2:** Colorless information and its suitability in urban underground space.

Achromatic color	Block	RGB	Suitability
Black		R:0 G:0 B:0	×
Dark gray		R = G = B<128	×
Light gray		R = G = B≥128	√
White		R:255 G:255 B:255	√

**Fig 11:**
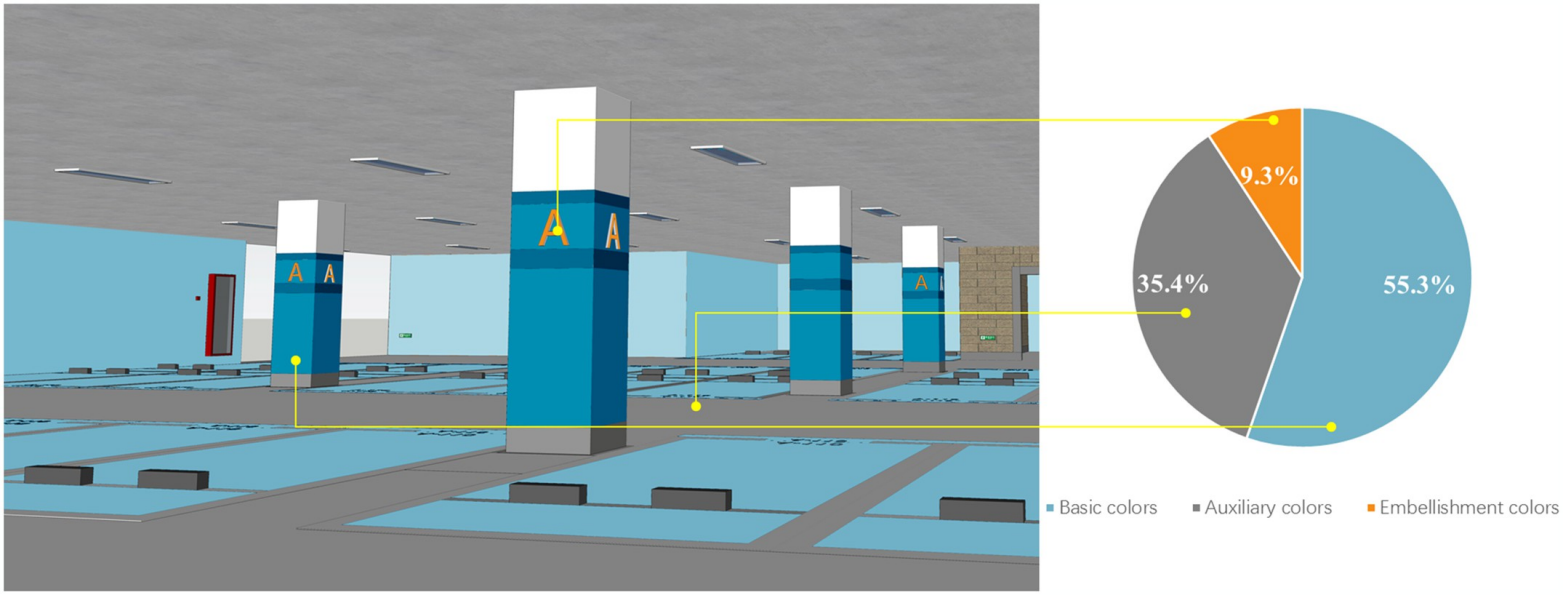
Color area ratio and scene display of theme colors based on golden section principle.

*(3) Theme colors*. In a large underground parking area, using a single color to define the theme may seem monotonous and may not effectively convey zoning and information. Utilizing a range of colors for both vertical and horizontal surfaces in such spaces improves overall color richness. Consequently, the theme color is defined by a primary color scheme perceived by users, including “basic color—auxiliary color—accent color.” The distribution of these colors is determined based on the golden section principle and applied to three color types under the theme to achieve a balanced color area proportion and establish a quantitative basis for a theme color grading system. The figure illustrates the color area ratios for various theme colors (Fig 11), showing significant aesthetic appeal when proportional relationships are applied to specific contexts. Importantly, safety colors, which fulfill special functional requirements and adhere to national standards, cannot be incorporated into the theme colors for underground spaces, except for designated uses.

*① Basic color chromatographic scheme*: The basic color, as indicated by its name, is applied to the largest surface areas, such as walls, columns, and parts of the floor in underground spaces. It is crucial for users to recognize the predominant color upon entering these areas. However, employing a single color throughout these surfaces can result in a rigid appearance and decrease the psychological perception of change. Color variation arises from the distance between colors: a smaller distance between colors results in a closer appearance and weaker contrast, while a larger distance enhances contrast. Consequently, based on the PCCS hue map for color coding and focusing primarily on surface elements, this study proposes two chromatographic schemes: a class color scheme and a complementary color scheme.

**Fig 12:**
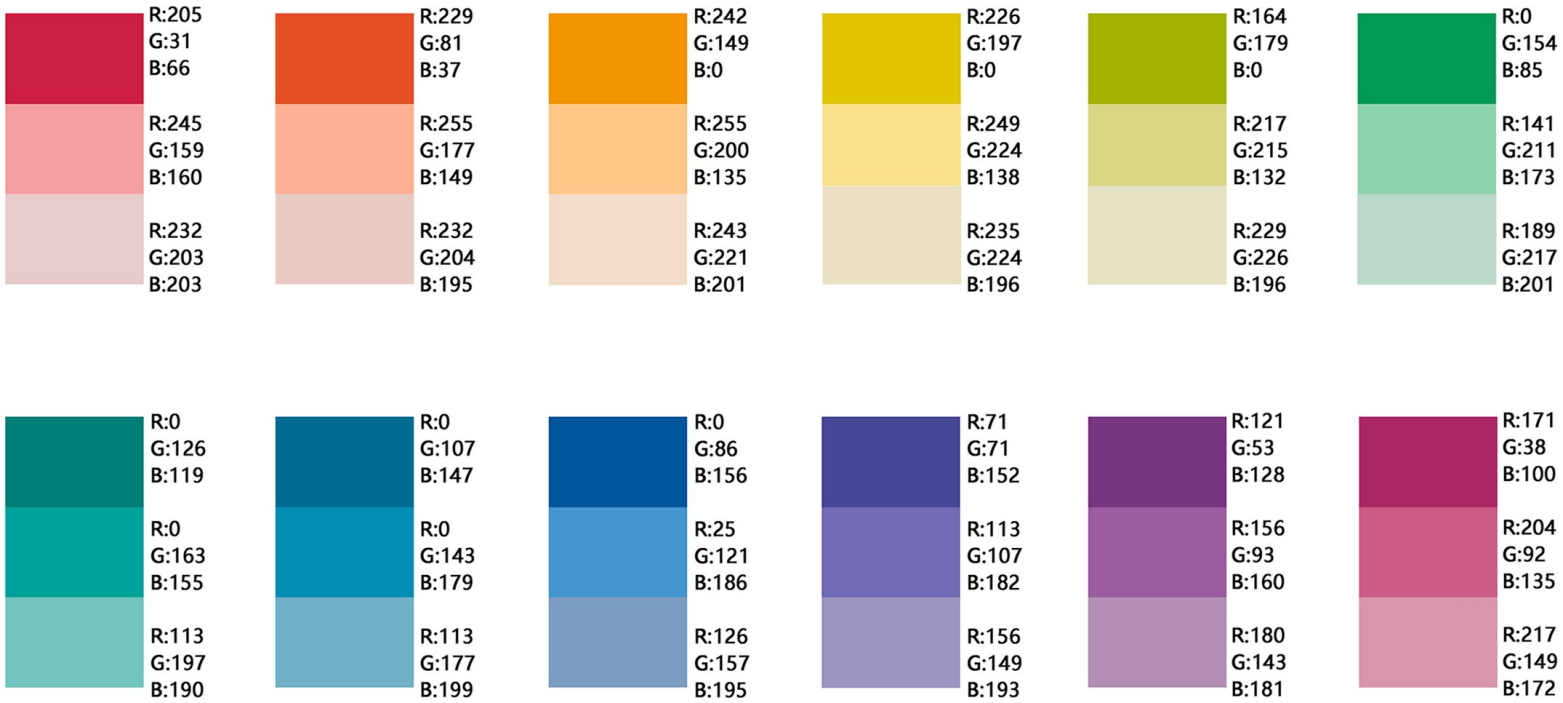
Basic color chromatographic scheme of urban underground space based on the principle of the homochromatic gradient.

——Class color scheme: The class color scheme is based on the principles of similarity and analogy, i.e., selecting colors with a difference of below 45° on the color wheel. When choosing colors from the same color wheel, minimal differentiation can occur due to the similarity of colors. Thus, variations in brightness and chromaticity must be considered. Colors may be chosen from the PCCS color wheel’s vivid tone (v), bright tone (b), light tone (lt), and pale tone (p) to create the basic color chromatography scheme (Fig 12). Specifically, for the warm color system in vivid tone (v), light tone (lt) and pale tone (p) colors can be selected to enhance differentiation, forming a warm color chromatographic scheme. For the cool color system within the vivid color ring, bright tone (b) and light tone (lt) with high color rendering can be selected to create a cool color chromatographic scheme. The class color scheme is designed to provide a harmonious, soft, gentle, and calming effect, making it suitable for underground parking spaces in residential areas, hospitals, libraries, schools, and other public service facilities.

**Fig 13:**
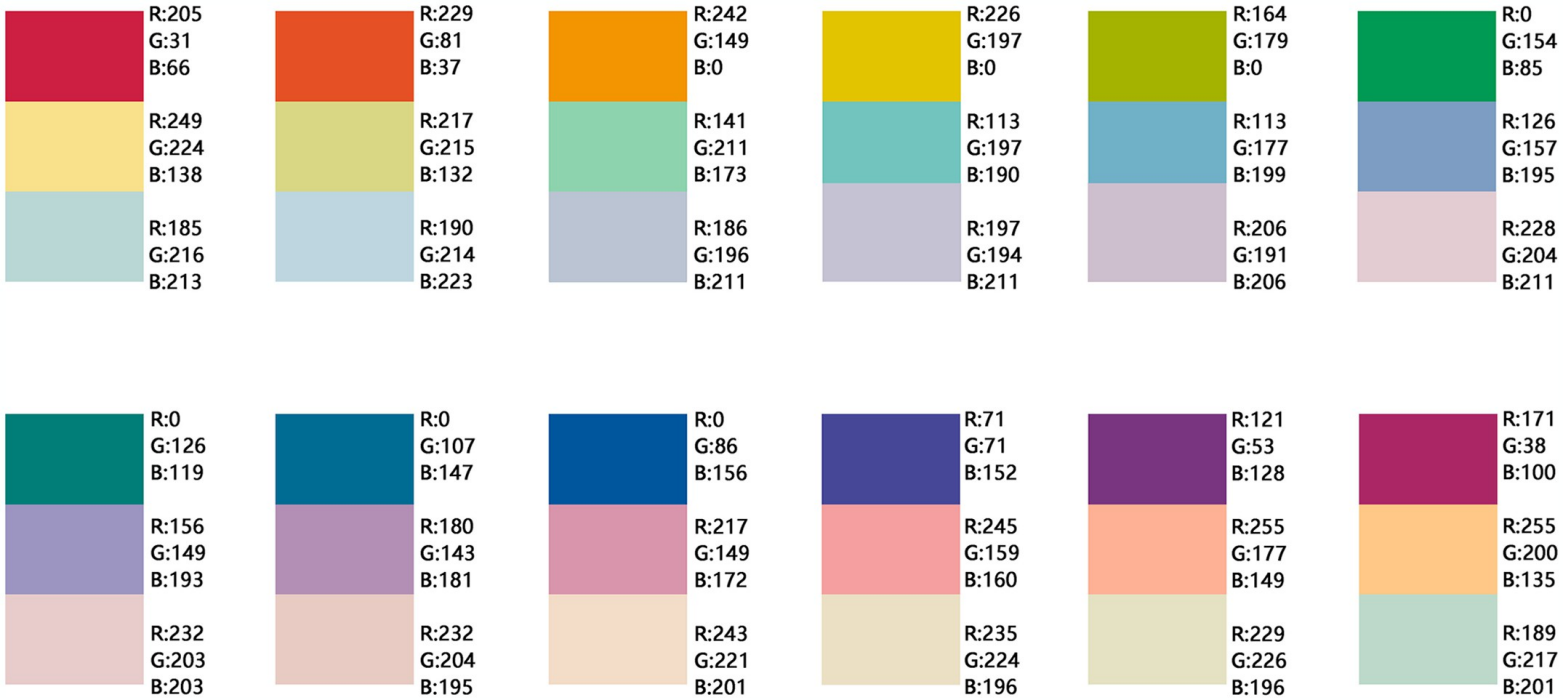
Basic color chromatographic scheme of urban underground space based on the diagonal principle of color rings.

——Complementary color scheme: The complementary color scheme is based on the diagonal complementarity principle of the PCCS color wheel, utilizing contrasting colors to form the basic color chromatography scheme (Fig 13). According to Eden’s theory of color harmonization [65], three colors are selected for harmonious application: a pale tone color (p) is used for the large-area basic color; the diagonal color of this pale tone in vivid tone (v) is chosen for key areas requiring emphasis and contrast; and the intermediate color between the two diagonal colors (in a clockwise direction) is used for smaller transitional areas. Light tones (lt) or bright tones (b) are recommended to illustrate transitions in lightness and chroma. This scheme provides flexibility in chromatogram selection and can create an eye-catching, vibrant, and visually rich environment. It is especially suitable for underground parking spaces in commercial complexes, children’s activity centers, sports facilities, and other public service facilities.

*② Auxiliary color chromatography scheme*: The auxiliary color is applied to secondary usage areas and must complement the basic color. It is used mainly in public access zones on the floor and ceiling of underground spaces and is essential for highlighting and differentiating the basic color. A significant contrast should exist between the auxiliary and basic colors, ensuring that the auxiliary color does not overshadow or alter the perception of the basic color. Typically, shades of gray, including variations between black and white, are suitable for this purpose. The color framework restricts the use of pure black and dark gray, so the acceptable range for auxiliary colors includes middle gray (mGy) to white (w). For basic colors with low brightness, matching the auxiliary colors to lower brightness levels of gray will help maintain overall brightness consistency within the underground spaces.

*③ Accent color chromatographic scheme*: The accent color pertains to the color applied to the wall, cylinder, ground, top pattern, logo, and pipeline. This color should contrast distinctly with the basic colors and auxiliary colors. In accordance with color usage principles, such as safety and prohibited colors, high chroma colors that offer eye-catching features are recommended. Vivid tones (v), strong tones (s), or other highly distinguishable hues can effectively transmit information through object use. For instance, in the context of a cylinder located in an underground parking facility within a class color scheme—whether cool or warm—the zoning logo (letter) should markedly differ from the basic color. Similarly, within complementary color scheme, sign colors must contrast sharply with both the cylinder’s base color and other areas to enhance the conveyed information.

**Fig 14:**
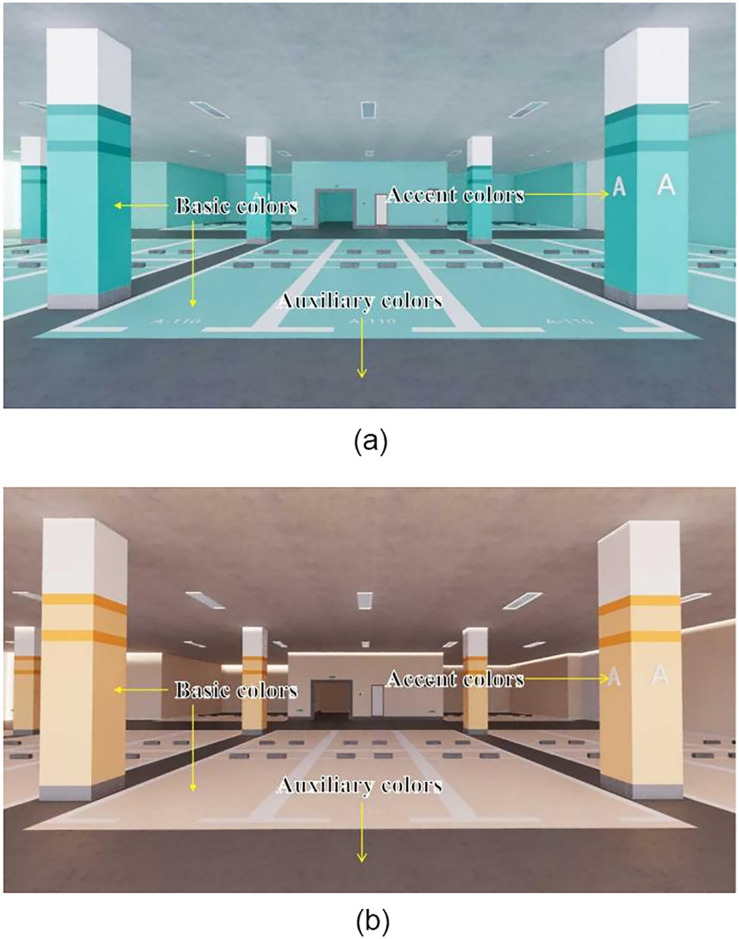
Application scenario display of the class color scheme in an underground parking lot.

**Fig 15:**
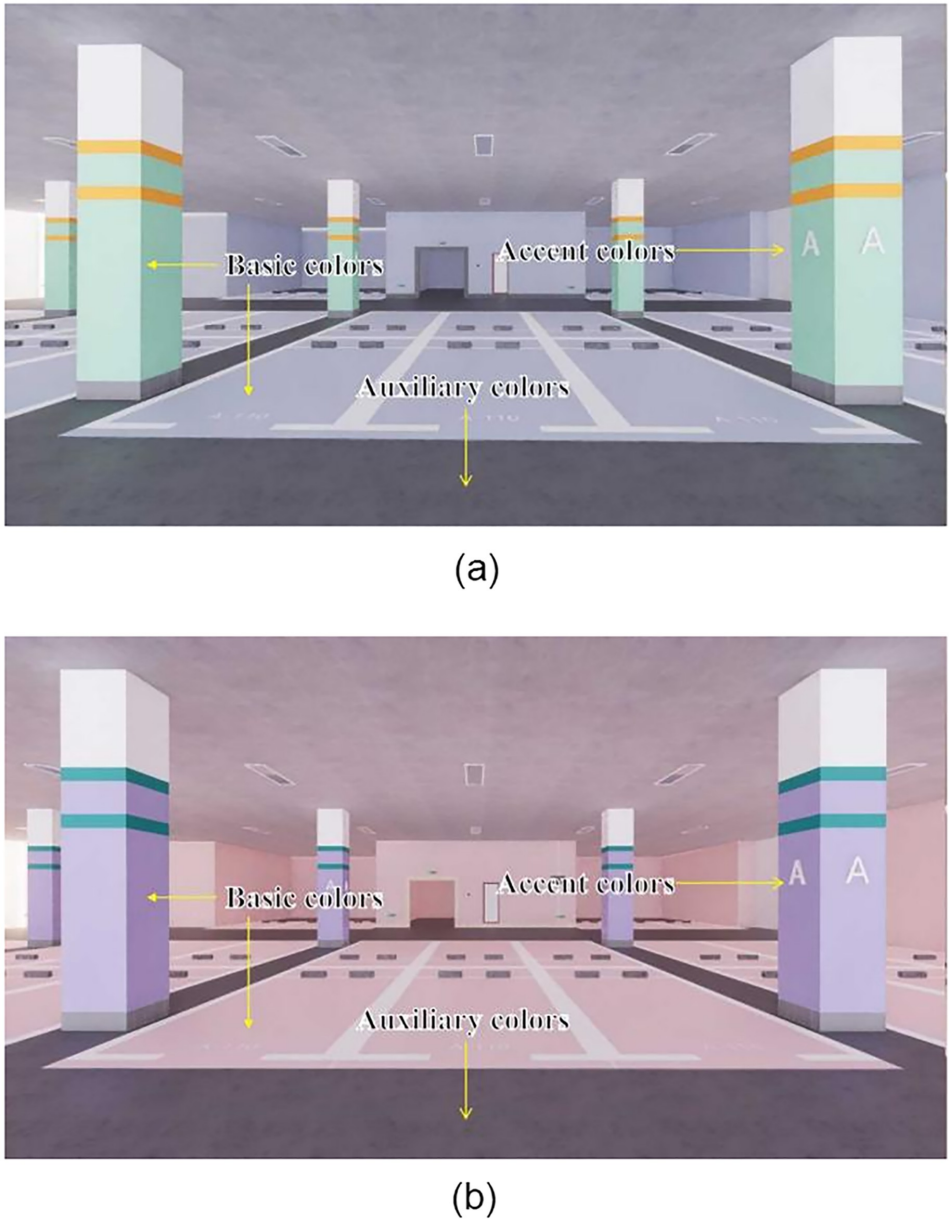
Application scenario of the complementary color scheme in an underground parking lot.

Based on the two basic chromatographic schemes described above, the color effect layout of underground spaces is exemplified. Sketch Up 2019 design software is employed to create a virtual three-dimensional model of an urban underground parking lot, incorporating various color carriers, including the top surface, cylinder surface, ground, and wall surfaces. Both the class color scheme and the complementary color scheme are utilized to conduct color tests, evaluating the overall application effect of these color schemes comprehensively. The scene displays for the class color and complementary color schemes are illustrated in Figs 14 and 15.

**Fig 16:**
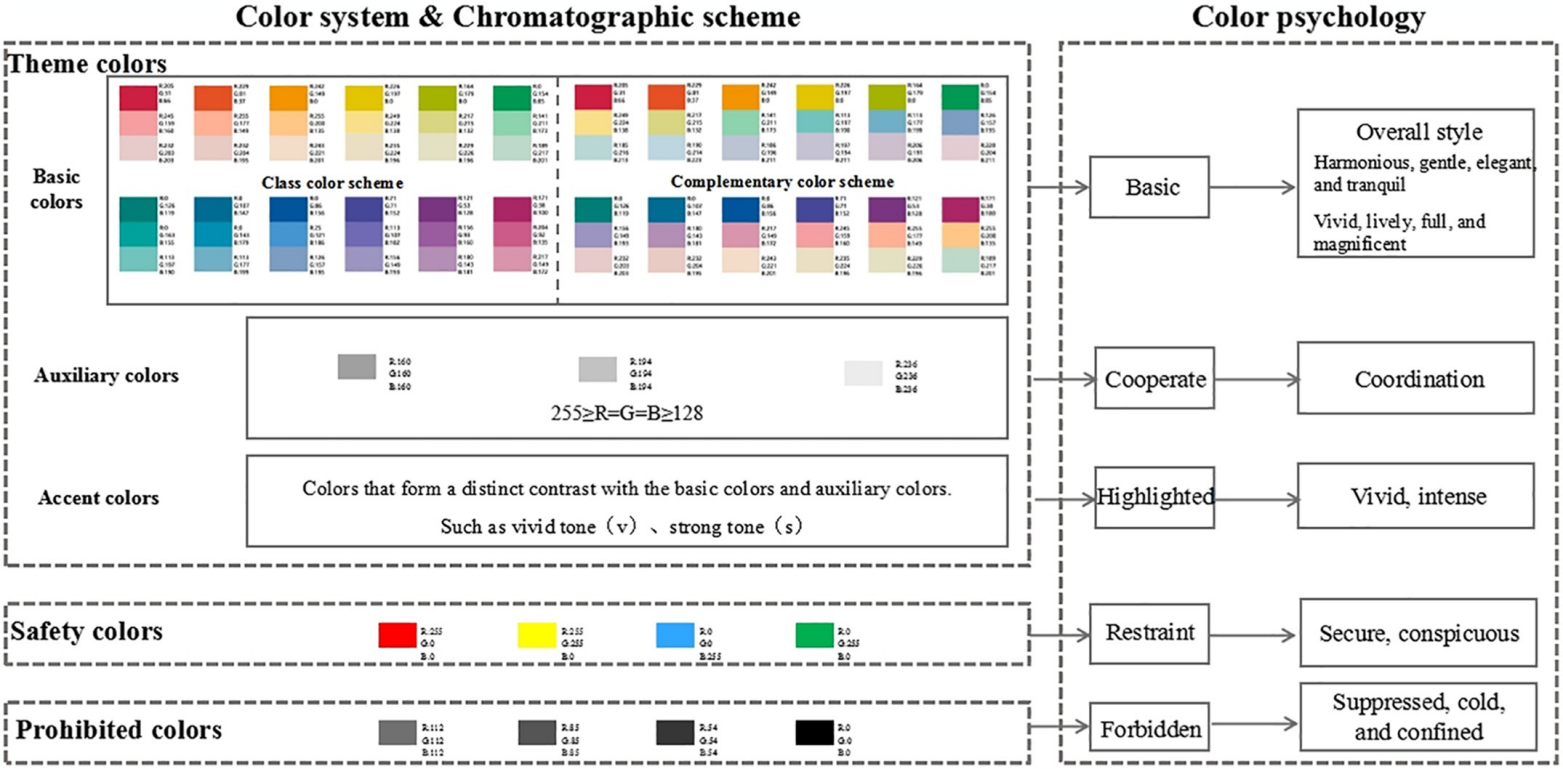
Chromatographic scheme library based on color psychology perception.

Therefore, based on theories such as color theory, color psychology theory, the golden section principle, and other relevant theories, the chromatographic scheme library depicted in Fig 16 has been established to align with public demands. Color application practices for urban underground parking spaces can be selected from this chromatographic scheme library.

### 5.2 Research contributions and limitations

This study investigated and analyzed the use of color in underground parking spaces, integrating urban planning and perceptual psychology. This research contributes to the understanding of color in urban underground environments. In contrast to prior studies, the focus of this research is on developing a framework for evaluating the color system of underground parking spaces based on psychological color perception. The specific theoretical contributions are as follows:

First, based on color theory and color psychology, an analytical framework for a color system is constructed following the sequence of “color field investigation—analysis of color influencing factors—color system construction—color application and control”. This framework has not been covered in previous research on underground parking spaces, yet it offers a practical structure for future color applications in such environments.

Second, unlike previous research on underground parking spaces, this study not only investigated color application with the understanding that “improving the quality of the underground space environment is crucial” but also extensively explored color psychology perception. The role and impact of color psychology were fully incorporated into the analytical framework, public psychological perception demands, chromatographic formulation, and other aspects. This approach offers a new perspective for related research.

Third, following the golden section principle, this study suggests a proportional relationship between color area and aesthetic composition to improve the public’s aesthetic experience in underground parking areas. Unlike previous research on color application, this study highlights the effectiveness of quantitative approaches in enhancing perceptual color coordination.

Fourth, based on the developed color system, a broader and more varied color selection is presented to offer guidance for applying a color system in urban underground parking areas.

In summary, this research advances the field of color planning for urban underground spaces by presenting an analytical framework for developing a color system for underground parking spaces and proposing a chromatographic scheme. This offers valuable insights for future applications of color analysis concepts in urban underground spaces and their practical implications.

However, this study has several limitations. The effect of color application is influenced by factors such as hue, proportion, and area, as well as the scale of the underground parking space, the base of the bearing object, the materials used, surface conditions, paint composition, and other related factors. Due to these limitations, future research should collaborate with experts in architectural engineering to apply the proposed color scheme in real-life settings and further refine it based on its color rendering effects.

Second, this study is based predominantly on field investigation techniques and theoretical summaries. The findings are primarily conveyed through perceptual cognition. At present, psychological research can employ laboratory methods for the quantitative analysis of perceptual cognition. Consequently, the subsequent step involves permitting public access to laboratories, where individuals can use physiological and psychological data collection equipment to examine the impact of different chromatic schemes on their psychological state. This will yield more specific color recommendations for managers of diverse underground environments.

## 6. Conclusion

The psychological perception of urban underground parking facilities is frequently negative due to characteristics such as darkness, enclosure, restricted visual field, and insufficient environmental guidance. Color, as a significant design element, has the potential to improve the user experience by creating a clearer and more comfortable underground environment. By strategically incorporating color into the design of these spaces, a connection between color, emotion, and the overall user experience can be established. This approach may ultimately foster the development of more welcoming and sustainable underground environments.

This paper employs a color psychology perspective to examine the use of color in underground parking facilities in typical Chinese cities. The research seeks to explore potential future applications of color systems in these settings and aims to develop a framework for analyzing color usage in urban underground parking spaces.

The main conclusions drawn from this research are as follows:

1. Following extensive field investigations in the initial stages of the research, several issues with urban underground parking spaces in China were identified, including “confusion in color application, insufficient color guidance functionality, and unclear color labeling in specific areas.” It is essential to implement effective color schemes to improve the current use of color in these underground environments.
2. This research has established a detailed framework for analyzing color systems, which offers significant practical value and feasibility: “color field investigation—analysis of color influencing factors—color system construction—color application and control”. The framework is based on a thorough review of current color usage. Important factors affecting color choice include the color elements and their relationships, the different types of underground parking spaces, and the color psychological perception needs of the public. The developed color system includes categories such as safety colors, prohibited colors, and theme colors, which are further divided into basic colors, auxiliary colors, and accent colors.
3. Based on field investigations, six public perception demands for underground parking spaces have been identified. The findings reveal varying levels of importance placed on these demands, with safety perception being the highest priority, followed by aesthetic perception, order perception, space scale perception, color distance perception, and angle and distance perception. To address these demands, the chromatographic scheme in the color system was informed by the “Safety Color” (GB 2893–2008), clarifying its application scope. Additionally, based on the golden section principle of aesthetics, proportional relationships of 55.3%, 35.4%, and 9.3% were proposed for the three colors within the theme color. Considering this proportional relationship and color system, a comprehensive chromatographic scheme for urban underground parking spaces is proposed to address public demands effectively.
4. Within the analytical framework of color, effective implementation and management are essential for creating a comfortable, aesthetically pleasing, and functional environment in underground parking spaces. To maximize the impact of color use, it should first be integrated with public policy characteristics and legal requirements in urban planning. Therefore, incorporating color planning content for urban underground parking spaces into city-level planning is recommended, along with developing detailed planning and design management methods for guidance. Additionally, a dedicated color management department should be established to oversee the implementation, adjustment, control, supplementation, and improvement of colors for long-term management. Public promotion and education regarding color management should also be conducted to encourage participation and ensure the continuity and stability of color usage.
